# Supplementary material for: Asynchronous magnetic resonance elastography: Shear wave speed reconstruction using noise correlation of incoherent waves
Source: Magn Reson Med. 2022 Oct 27;89(3):990–1001. doi: 10.1002/mrm.29502 (PMC9792433; doi:10.1002/mrm.29502)
Supplement: Supplementary file 1 — DATA S1 MATLAB code used to generate the numerical simulations of Figure 1 [file MRM-89-990-s002.zip › k-Wave/helpfiles/checkFactors.html]

checkFactors :: Functions (k-Wave)


# checkFactors

Return the maximum prime factor for a range of numbers.

## Syntax

```
checkFactors(min_number, max_number)
```

## Description

`checkFactors` loops through the given range of numbers and finds the numbers with the smallest maximum prime factors. This allows suitable grid sizes to be selected to maximise the speed of the FFT (this is fastest for FFT lengths with small prime factors). The output is printed to the command line, and a plot of the factors is generated.

For example, running the code `checkFactors(100, 200)` produces the folling output.

```
Numbers with a maximum prime factor of 2
128
Numbers with a maximum prime factor of 3
108  144  162  192
Numbers with a maximum prime factor of 5
100  120  125  135  150  160  180  200
Numbers with a maximum prime factor of 7
105  112  126  140  147  168  175  189  196
Numbers to avoid (prime numbers)
101  103  107  109  113  127  131  137  139  149  151  157  163  167  173  179  181  191  193  197  199
```

## Inputs

|  |  |
| --- | --- |
| `min_number` | integer specifying the lower bound of values to test |
| `max_number` | integer specifying the upper bound of values to test |

## See Also

`fft`, `factor`
